# Patterns of prescription, over-the-counter, and herbal medication use among pregnant women in Buraydah, Saudi Arabia: a cross-sectional study

**DOI:** 10.3389/fphar.2025.1732619

**Published:** 2026-01-02

**Authors:** Masaad S. Almutairi, Mustafa S. Saeed, Hessa Alowias, Afnan Alharbi, Faris S. Alnezary, Omar A. Almohammed

**Affiliations:** 1 Department of Pharmacy Practice, College of Pharmacy, Qassim University, Buraidah, Qassim, Saudi Arabia; 2 Department of Pharmacy Practice, College of Pharmacy, Taibah University, Madinah, Saudi Arabia; 3 Department of Clinical Pharmacy, College of Pharmacy, King Saud University, Riyadh, Saudi Arabia; 4 Pharmacoeconomics Research Unit, College of Pharmacy, King Saud University, Riyadh, Saudi Arabia

**Keywords:** drug safetey, pharmacovigilance, pregnancy, public health, Saudi Arabia

## Abstract

**Background:**

Medication use during pregnancy is a significant public health consideration due to potential risks that certain medication can cause to both the mother and the developing fetus. Understanding regional patterns of medication use is crucial for targeted patient counseling and pharmacovigilance. This study aimed to characterize the prevalence, types, and predictors of prescription, over-the-counter (OTC), and herbal medication use among pregnant women in Buraydah, Saudi Arabia.

**Methods:**

A cross-sectional survey was conducted among 407 pregnant women receiving antenatal care at the Maternity and Children Hospital in Buraydah. Data on demographics, clinical characteristics, and medication use were collected using a structured questionnaire and analyzed using descriptive statistics and multivariable logistic regression.

**Results:**

Medication use was found to be highly prevalent. Almost all participants (98.3%) used pregnancy-related supplements, with folic acid being the most common. Prescription medication for short-term conditions was reported by 43.5% of women, with antibiotics (62.1%) and analgesics (42.9%) being the most frequent. In addition 19.4% of them used medications for chronic conditions, primarily hormone replacement therapy (44.3%) for hypothyroidism. OTC analgesics were used by half (50%) of the 36.9% of women taking nonprescription drugs; 19.4% of them used herbal medicine, with ginger being the most common (44.7%) ingredient for nausea. Further, women in their second (OR: 6.75) and third (OR: 8.71) trimesters were more likely to use short-term prescription medications compared to those in their first trimester.

**Conclusion:**

Medication and supplement use was found to be widespread among the studied cohort of pregnant women. The findings emphasize the need for enhanced pharmacovigilance and proactive patient counseling by healthcare providers, particularly pharmacists, to ensure maternal and fetal safety.

## Introduction

Medication use and pregnancy are likely to coexist since women constitute one-third of the global drug user population, and a large proportion of these women are of reproductive age (Mburu et al., 2020). Studies conducted in North and South America, Australia, and Europe have found that most pregnant women there take at least one medication (Engeland et al., 2018). Approximately 70% of pregnant women often use prescription drugs. These drugs treat acute and chronic diseases, as well as pregnancy-related conditions (Hudson et al., 2023). Moreover, pregnancy-related illnesses that require ongoing or intermittent treatment may also be one of the reasons for medication use (Navaro et al., 2018).

A study conducted in the US found that 95% of pregnant women utilize more than three over-the-counter (OTC) and prescription medications. Gastrointestinal or antiemetic agents followed by antibiotics were found to be most frequently used for nausea, vomiting, and urinary tract infections (UTIs) (Haas et al., 2018). A Kenyan study identified two main reasons for taking medications during pregnancy: managing unplanned pregnancy stress and treating withdrawal through the continuous use of drugs (Mburu et al., 2020). An Ethiopian study conducted in 2020 revealed the most frequently used medications during pregnancy, which included anti-anemics followed by analgesics and anti-infectives (Ayele et al., 2020). Moreover, a French study found that almost 90% of the included patients were using medications during their pregnancy (Bérard et al., 2019). In addition, a study in Norway found medication use during pregnancy in 60% of the participants (Engeland et al., 2018).

Further, the level of knowledge on the risk of using medications during pregnancy has been documented across different studies. In Italy, a study found that pregnant women belonging to a high socioeconomic status had a high level of knowledge on the risk of using medications during pregnancy (Navaro et al., 2018). In addition, in Nepal, the occupation of the women and their level of education was found to be significantly associated with the level of knowledge regarding their medication use (Devkota et al., 2017). In the UK, three out of ten pregnant avoided using medication during pregnancy due to their belief that medication was overused and not very beneficial in treating health conditions (Twigg et al., 2016). Furthermore, in Australia, a high health literacy was found to be strongly associated with participant’s skills of seeking more information on complementary medicine products from healthcare providers during pregnancy (Barnes et al., 2019).

A study conducted in Bangladesh clarified that pregnant women preferred herbal medicine due to its safety, accessibility, affordability, and efficiency as well as due to family tradition and discontentment with contemporary medicine (Ahmed et al., 2018). Pregnant women worldwide frequently use herbal products, such those containing ginger and garlic as well as herbal teas for common ailments (such as UTIs, colds, flu, nausea, and vomiting) (Illamol et al., 2020). Further, pregnancy necessitates a macronutrient-balanced diet—as protein requirements increase, particularly in the third trimester—thus promoting production while limiting significant fat consumption (Kocyłowski et al., 2018). Most pregnant women in the United States have been found to utilize at least one nutritional supplement at some point throughout their pregnancy. Pregnant women may utilize dietary supplements to improve their nutrient consumption because their intake of foods does not meet their actual needs. Increased caloric and nutrient intakes are required to support the physiological needs of the developing fetus and mother, particularly folate, iron, iodine, and copper. Thus, dietary supplements should be carefully planned. In addition, it has been found that pregnant women aged 20–34 years in their first trimester or from lower-income families were less likely to use supplements than their peers (Bailey et al., 2019; Jun et al., 2020).

Maternal immunization is a novel public health strategy that protects both the mother and her fetus or newborn from infections in the second or third trimester. Certain countries have implemented routine antenatal vaccinations, which are currently restricted to pertussis, tetanus, diphtheria, inactivated polio, and seasonal influenza vaccines (Sebghati and Khalil, 2021). Vaccinations against influenza, pertussis, and tetanus are strongly recommended for safe use during pregnancy. Among the others that are being developed are vaccines against the respiratory syncytial virus (RSV), group B *Streptococcus* (GBS), and cytomegalovirus (CMV) (Maertens et al., 2020).

In Saudi Arabia, medications use during pregnancy has been evaluated in several studies. A recent study conducted in Riyadh found that 76% of the participants reported using medications during their current pregnancy, while 86.3% used prescribed medications (Alyami et al., 2023). In addition, a study that included 760 participants found that almost 40% reported using medication during their pregnancy (Zaki et al., 2014). Despite health promotion campaigns, the level of medication utilization was scattered among different populations. The objectives of this study were to evaluate the patterns of prescription, OTC, and herbal medication use among pregnant women in Saudi Arabia.

## Methods

### Study design, population, and setting

A cross-sectional questionnaire-based study was conducted from October 22, 2023, to April 15, 2024, to identify the most frequently used medications or supplements during pregnancy and the reason for taking them. The survey was conducted in the Maternity and Children Hospital in Buraydah, Qassim region of the Kingdom of Saudi Arabia. A convenience sample of pregnant women receiving antenatal care services at the outpatient clinics of the Maternity and Children Hospital in Buraydah were recruited during the study period. We excluded pregnant women who were unable to respond due to physical or mental impairments as well as very ill pregnant women. Eligible and willing participants were included until the target sample size was met. This study included human subjects, and the ethical approval for this study proposal was obtained from the Qassim University Research and Ethics Committee, Saudi Arabia (No: 607/45/4934).

### Study instrument

The study was conducted using a questionnaire that was adopted from previous literature (Haas et al., 2018; Lupattelli et al., 2014; Van Gelder et al., 2018). The questionnaire is composed of two sections. The first section records the demographic data of the pregnant women, such as their age, number of prior children, level of education, etc. The second section records the characteristics of the utilization of prescription medications (short and chronic usage), nonprescription medications, pregnancy related medications, and herbals; this section is used for the evaluation of the trend in types of drugs and products used by pregnant women (Supplementary Appendix A).

To ensure validity, the questionnaire was developed through a multistep process. Although this instrument was adopted from previously validated and published instruments, it was further revised and culturally modified by a team of experts in pharmacy practice, pharmacoepidemiology, and social pharmacy. Then, it was further revised by two physicians from the gynecology/maternal health field who routinely provide care to pregnant women. After multiple rounds of revisions, all experts approved the instrument (Supplementary Appendix A), which was then pilot tested among 20 pregnant women to ensure the clarity of the items on the questionnaire; no changes were made at the pilot testing stage. This process ensured that each item was comprehensible, contextually appropriate, and aligned with the study aims. While no psychometric scales were constructed, the questionnaire demonstrated face and content validity through expert reviews and pilot testing, which supports its suitability for the purposes of this study.

### Statistical analysis

Descriptive statistics were applied as appropriate to analyze participants characteristics in the first section, such as age, place of residence, education level, occupation, gestational age, gravidity, parity, husband’s education level, husband’s occupation, and husband’s income. In the second section of the questionnaire, drug use characteristics were analyzed, including the use of medication for acute/short-term illnesses, medication use for chronic/long-term disorders, and OTC/herbal medication use. Logistic regression analyses were performed to examine the association between participants’ characteristics, which was the independent covariate in the regression model (age, place of residence, education level, occupation, gestational age, gravidity, parity, husband’s education level, husband’s occupation, and husband’s income), and the participants’ use of medications for acute/short-term illnesses or chronic/long-term disorders as well as the use of OTC medications or herbal products as the dependent or outcome variables in the models. Assuming that 50% of pregnant women would report using at least one medication or supplement during pregnancy, with a 95% confidence level and a 5% margin of error, the estimated sample size was calculated to be 385 participants. A p-value of less than 0.05 was considered significant. Odds ratios (ORs) with 95% confidence intervals (CIs) were calculated to quantify associations from the logistic regression models. The Statistical Package for Social Sciences (SPSS) software version 29 was used to analyze the data.

## Results

### Participants characteristics

In this study, 457 pregnant women were invited to participate, with 407 providing their consent to participate in the survey, which resulted in an 89% response rate. The final analysis incorporated data from all these 407 pregnant women. The current study indicates that 37.3% of the participants fall within the age groups of 30–34 years, and 28.3% were in the age groups of 25–29 years. The results also indicate that 92.9% of the participants were residing in urban areas. In terms of educational level, 54.5% of the participants held a university or college degree and 75.2% of the participants were housewives. In addition, 71% of the participants were in the third trimester of their pregnancy and 51.1% of the participants had more than two previous pregnancies. With regard to the husband’s educational level, the husbands of 34.9% of the participants’ had at least a high school education and those of 48.9% had university/college degrees; Moreover, almost two-thirds of the husbands were government employees. It was also found that 83.8% of the husbands fall into the lower middle-income category (Table 1).

**TABLE 1:** Characteristics of study participants (n = 407).

Characteristics and categories		n (%)
Age (years)	<25	41 (10.1)
	25–29	115 (28.3)
	30–34	152 (37.3)
	≥35	99 (24.3)
Place of residence	Urban	378 (92.9)
	Rural	29 (7.1)
Education level	Illiterate	20 (4.9)
	Primary school	10 (2.5)
	Intermediate school	27 (6.6)
	High school	108 (26.5)
	University/College	222 (54.5)
	Postgraduate studies	20 (4.9)
Occupation	Housewife	306 (75.2)
	Government employee	51 (12.5)
	Private employee	23 (5.7)
	Student	20 (4.9)
	Other	7 (1.7)
Gestational age (weeks)	Third trimester	289 (71.0)
	Second trimester	91 (22.4)
	First trimester	27 (6.6)
Gravidity (no. of previous pregnancies)	One	132 (32.4)
	Two	67 (16.5)
	More than two	208 (51.1)
Parity (no. of previous live births)	None	125 (30.7)
	Two	107 (26.3)
	More than two	175 (43.0)
Husband’s occupation	Government employee	275 (67.6)
	Private employee	73 (17.9)
	Self-employee	59 (14.5)
Husband’s educational level	Illiterate	6 (1.5)
	Primary school	7 (1.7)
	Intermediate school	22 (5.4)
	High school	142 (34.9)
	University/College	199 (48.9)
	Postgraduate studies	31 (7.6)
Husband’s income	Low income	34 (8.4)
	Lower middle income	341 (83.7)
	Upper middle income	32 (7.9)

Data are presented as the number of patients (%).

### Using medication for short-term

**FIGURE 1:**
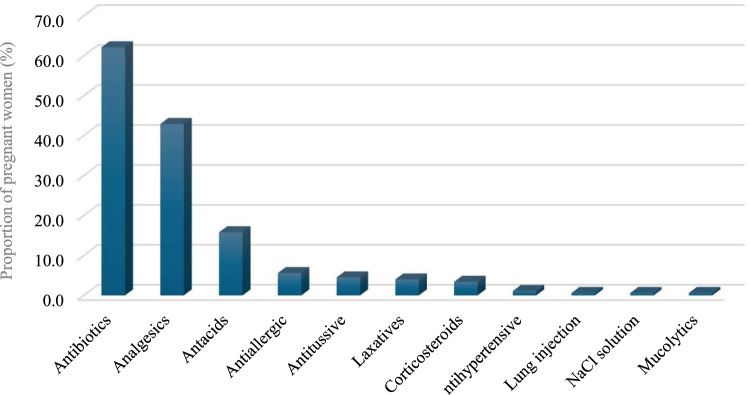
Medications used for short term.

A total of 177 participants (43.5%) were found to be using medications for short-term conditions, whereas the remaining 56.5% of the participants did not use any prescription medication for short-term conditions. This study reveals that antibiotics were the most prescribed medication (62.1%) for short-term conditions among the participants, mostly for UTI, followed by analgesics (42.9%), with a short duration being the most frequently reported by participants (81.4%). These data are depicted in Table 2 and Figure 1.

**TABLE 2:** Use of prescription medications for short-term conditions (n = 177).

Items and responses	n (%)
Did you use any prescription medication for short-term? (n = 407)	
No	230 (56.5)
Yes	177 (43.5)
The period of using medication for short-term (n = 177)	
1 Month or less	144 (81.4)
2 Month	3 (1.7)
3 Month	11 (6.2)
When needed	19 (10.7)
Indication to use medication for short-term period (n = 177)^a^	
Urinary tract infections	72 (40.7)
Acidity (heartburn)	33 (18.7)
Fever	30 (16.9)
Headaches	23 (13.0)
Cold	14 (7.9)
Vaginal infections	13 (7.3)
Sore throat	12 (6.8)
Cough	10 (5.6)
Allergy	9 (5.1)

^a^ Participant could report less or more than one indication; therefore, percentages may not amount to 100%.

### Using medication for chronic conditions

**FIGURE 2:**
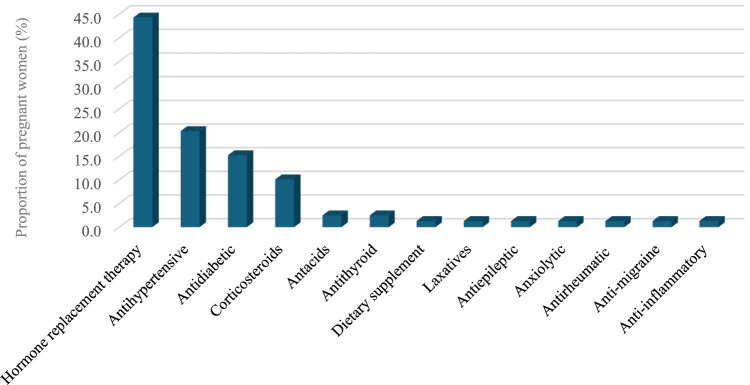
Medications used for chronic conditions.

A relatively small proportion of participants (19.4%) reported using prescription medication for chronic conditions (Table 3; Figure 2). Medicines for hormone replacement therapy were the most frequently prescribed medication for chronic conditions (44.3%), followed by antihypertensive and antidiabetic medications (20.3% and 15.2%, respectively). With regard to chronic conditions, hypothyroidism, hypertension, and diabetes mellitus were the most reported chronic conditions related to these medications (44.3%, 20.3%, and 15.2%, respectively).

**TABLE 3:** Use of prescription medication for chronic conditions (n = 79).

Items and responses	n (%)
Did you use any prescription medication for chronic conditions (n = 407)	
No	328 (80.6)
Yes	79 (19.4)
Indication for chronic conditions medications (n = 79)^a^	
Hypothyroidism	35 (44.3)
Hypertension	16 (20.3)
Diabetes mellitus	12 (15.2)
Asthma	6 (7.6)
Heartburn and acidity	2 (2.5)
Hyperthyroidism	2 (2.5)
Lupus	2 (2.5)
Gestational diabetes	2 (2.5)
Epilepsy	1 (1.3)
Osteoporosis	1 (1.3)
Polycystic ovary syndrome	1 (1.3)
Immunodeficiency	1 (1.3)
Rheumatism	1 (1.3)
Migraines	1 (1.3)
Constipation	1 (1.3)
Anxiety	1 (1.3)
The period of using medications for chronic conditions (n = 79)	
Throughout the 9 months of pregnancy	52 (65.9)
When needed	3 (3.8)
1 month or less	3 (3.8)
2 months	1 (1.3)
3 months	6 (7.6)
4 months	2 (2.5)
5 months	2 (2.5)
6 months	5 (6.3)
7 months	5 (6.3)

^a^ Participant could report less or more than one indication; therefore, percentages may not amount to 100%.

### Using pregnancy-related medications, nonprescription medications and herbal medicine

**FIGURE 3:**
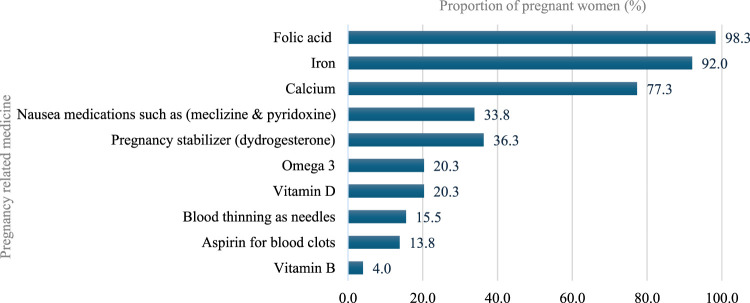
Pregnancy-related medications use during pregnancy.

**FIGURE 4:**
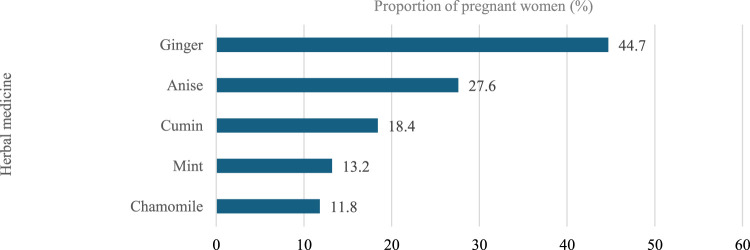
Herbal medicine used during pregnancy.

**FIGURE 5:**
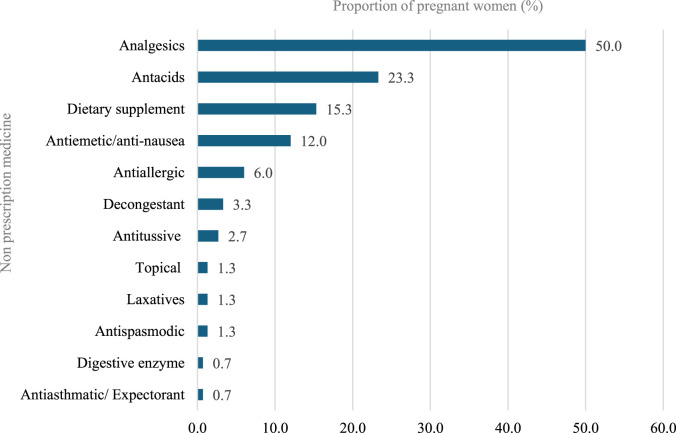
Non-prescription medications use during pregnancy.

Most participants (98.3%) reported using pregnancy-related drugs, such as vitamins and minerals, with folic acid being the most common (98.3%), followed by iron (92.0%), calcium (77.3%), meclizine and pyridoxine (33.8%), dydrogesterone (36.3%), Omega (20.3%), and Vitamin D (20.3%). With regard to nonprescription medications, 150 (36.9%) participants reported using nonprescription medications during pregnancy. Analgesics were the most used (50%), followed by antacids (23.3%), dietary supplements (15.3%), and antiemetic/anti-nausea (12.0%). Further, the most common indications for using nonprescription medications by these participants during pregnancy were headache (28.7%), acidity (18.7%), and nausea (12.7%). The most frequent duration of using nonprescription medications was as needed (49.3%), followed by 1 month or less 29.3% (Figures 3–5).

Further, 19.4% of the participants reported using herbal medicine during their pregnancy. Herbal medicine containing ginger was the most commonly used during pregnancy among the participants (44.7%), followed by anise (27.6%). Moreover, the most commonly reported indications for using herbal medicine during pregnancy were nausea (42.1%), flatulence (13.2%), and facilitating childbirth (15.8%). The most frequent duration of using herbal medicines was as needed (56.6%), followed by 1 month or less (31.6%).

### Predictors of medications use during pregnancy

The multivariable logistic regression analysis (Tables 4, 5) identified several significant predictors for short-term prescription medication use. The use of prescription medications for short term during pregnancy was significantly higher in pregnant mothers during their second or third trimesters of gestational age compared to those in their first trimester (OR = 6.75; 95% CI 1.82–25; OR = 8.71, 95% CI 2.45–30.93, respectively). In contrast, women who gave birth more than twice were less likely to use prescription medications in the short term (OR = 0.31, 95% CI 0.12–0.82) compared to women with no previous birth experience. Moreover, women who were married to an employee of a private organization were less likely to use prescription medication in the short-term during their pregnancy compared to wives of government employees (OR = 0.54, 95% CI 0.30–0.97). Further analysis for prescription medications (chronic use), nonprescription, and herbal medication during pregnancy revealed no significant factors associated with their consumption.

**TABLE 4:** Multivariable logistic regression analysis of predictors for medication uses during pregnancy.

Characteristics and categories		Medications for short-term			Medications for chronic conditions		
		No	Yes	OR (95% CI)	No	Yes	OR (95% CI)
Age (years)	<25	26 (11.3)	15 (8.5)	Ref	37 (11.3)	4 (5.1)	Ref
	25–29	37 (31.7)	42 (23.7)	0.85 (0.36–1.98)	98 (29.9)	17 (21.5)	0.82 (0.23–2.90)
	30–34	81 (35.2)	71 (40.1)	1.35 (0.57–3.23)	120 (36.6)	32 (40.5)	1.30 (0.37–4.52)
	≥35	50 (21.7)	49 (27.7)	1.60 (0.61–4.19)	73 (22.3)	26 (32.9)	2.22 (0.59–8.34)
Place of residence	Urban	214 (93)	164 (92.7)	Ref	305 (93)	73 (92.4)	Ref
	Rural	16 (7)	13 (7.3)	1.08 (0.46–2.49)	23 (7)	6 (7.6)	1.00 (0.35–2.82)
Education level	Illiterate	13 (5.7)	7 (4)	Ref	17 (5.2)	3 (3.8)	Ref
	Primary school	4 (1.7)	6 (3.4)	2.34 (0.41–13.2)	9 (2.7)	1 (1.3)	0.79 (0.05–11.2)
	Intermediate school	13 (5.7)	14 (7.9)	1.85 (0.49–6.99)	25 (7.6)	2 (2.5)	0.55 (0.07–4.32)
	High school	67 (29.1)	41 (23.2)	0.93 (0.28–3.03)	89 (27.1)	19 (24.1)	1.82 (0.35–9.46)
	University/College	122 (53)	100 (56.5)	1.20 (0.37–3.87)	171 (52)	51 (64.6)	2.99 (0.58–15.3)
	Postgraduate studies	11 (4.8)	9 (5.1)	1.03 (0.20–5.18)	17 (5.2)	3 (3.8)	1.71 (0.17–16.3)
Occupation	Student	13 (5.7)	7 (4)	Ref	20 (6.1)	0	NAE
	Housewife	176 (76.5)	130 (73.4)	1.38 (0.47–4.03)	246 (75)	60 (75.9)	NAE
	Government employee	24 (10.4)	27 (15.3)	1.90 (0.55–6.55)	41 (12.5)	10 (12.7)	NAE
	Private employee	12 (5.2)	11 (6.2)	1.51 (0.38–5.88)	16 (4.9)	7 (8.9)	NAE
	None of the above	5 (2.2)	2 (1.1)	0.71 (0.10–5.07)	5 (1.5)	2 (2.5)	NAE
Gestational age (weeks)	First trimester	24 (10.4)	3 (1.7)	Ref	18 (5.5)	9 (11.4)	Ref
	Second trimester	51 (22.2)	40 (22.6)	**6.75 (1.82**–**25.0)**	69 (21)	22 (27.8)	0.78 (0.28–2.14)
	Third trimester	155 (67.4)	134 (75.7)	**8.71 (2.45**–**30.9)**	241 (73.5)	48 (60.8)	0.46 (0.18–1.16)
Gravidity	One	79 (34.3)	53 (29.9)	Ref	111 (33.8)	21 (26.6)	Ref
	Two	40 (17.4)	27 (15.3)	1.59 (0.73–3.46)	50 (15.2)	17 (21.5)	1.01 (0.40–2.55)
	More than two	111 (48.3)	97 (54.8)	2.40 (0.98–5.85)	167 (50.9)	41 (51.9)	0.58 (0.19–1.72)
Parity	None	70 (30.4)	55 (31.1)	Ref	109 (33.2)	16 (20.3)	Ref
	Two	64 (27.8)	43 (24.3)	0.51 (0.25–1.06)	79 (24.1)	28 (35.4)	2.59 (1.06–6.34)
	More than two	96 (41.7)	79 (44.6)	**0.31 (0.12**–**0.82)**	140 (42.7)	35 (44.3)	2.47 (0.74–8.27)
Husband’s occupation	Government employee	146 (63.5)	129 (72.9)	Ref	220 (67.1)	55 (69.6)	Ref
	Private employee	46 (20)	27 (15.3)	**0.54 (0.30**–**0.97)**	60 (18.3)	13 (16.5)	1.05 (0.49–2.26)
	Self-employee	38 (16.5)	21 (11.9)	0.63 (0.30–1.30)	48 (14.6)	11 (13.9)	0.94 (0.38–2.33)
Husband’s educational level	Illiterate	4 (1.7)	2 (1.1)	Ref	4 (1.2)	2 (2.5)	Ref
	Primary school	4 (1.7)	3 (1.7)	0.99 (0.08–11.8)	5 (1.5)	2 (2.5)	0.32 (0.02–5.35)
	Intermediate school	13 (5.7)	9 (5.1)	0.65 (0.07–5.68)	20 (6.1)	2 (2.5)	0.10 (0.01–1.47)
	High school	79 (34.3)	63 (35.6)	0.96 (0.13–7.12)	116 (35.4)	26 (32.9)	0.13 (0.01–1.27)
	University/College	111 (48.3)	88 (49.7)	0.86 (0.11–6.66)	157 (47.9)	42 (53.2)	0.16 (0.02–1.71)
	Postgraduate studies	19 (8.3)	12 (6.8)	0.86 (0.09–7.57)	26 (7.9)	5 (6.3)	0.17 (0.01–2.21)
Husband’s income	Low income	21 (9.1)	13 (7.3)	Ref	28 (8.5)	6 (7.5)	Ref
	Lower middle income	187 (81.3)	154 (87)	0.98 (0.40–2.43)	274 (83.5)	67 (84.8)	0.81 (0.25–2.63)
	Upper middle income	22 (9.6)	10 (5.6)	0.46 (0.13–1.56)	26 (7.9)	6 (7.6)	0.74 (0.16–3.34)

Numbers were presented as frequency (%); NAE: not able to estimate.

Bold: Statistical significance.

**TABLE 5:** Predictors of using Non-prescription medications and herbal medicine during pregnancy.

Characteristics and categories		Non-prescription medications			Herbal medicine		
		No	Yes	OR (95% CI)	No	Yes	OR (95% CI)
Age (years)	<25	27 (10.5)	14 (9.3)	Ref	36 (10.9)	5 (6.6)	Ref
	25–29	82 (31.9)	33 (22)	0.90 (0.38–2.12)	96 (29)	19 (25)	1.76 (0.52–5.92)
	30–34	90 (35)	62 (41.3)	1.36 (0.56–3.29)	121 (36.6)	31 (40.8)	2.79 (0.78–9.95)
	≥35	58 (22.6)	41 (27.3)	1.26 (0.47–3.39)	78 (23.6)	21 (27.6)	3.29 (0.83–12.9)
Place of residence	Urban	234 (91.1)	144 (96)	Ref	308 (93.1)	70 (92.1)	Ref
	Rural	23 (8.9	6 (4)	0.40 (0.14–1.07)	23 (6.9)	6 (7.9)	1.76 (0.61–5.06)
Education level	Illiterate	17 (6.6)	3 (2)	Ref	19 (5.7)	1 (1.3)	Ref
	Primary school	9 (3.5)	1 (0.7)	0.54 (0.04–6.66)	4 (1.2)	6 (7.9)	21.8 (1.71–278.6)
	Intermediate school	17 (6.6)	10 (6.7)	3.06 (0.62–14.9)	24 (7.3)	3 (3.9)	2.13 (0.17–25.4)
	High school	59 (23)	49 (32.7)	4.24 (1.03–17.65)	91 (27.5)	17 (22.4)	3.12 (0.33–29.1)
	University/College	140 (54.5)	82 (54.7)	3.36 (0.81–14)	179 (54.1)	43 (56.6)	4.94 (0.53–45.6)
	Postgraduate studies	15 (5.8)	5 (3.3)	1.63 (0.25–10.6)	14 (4.2)	6 (7.9)	6.38 (0.54–80.8)
Occupation	Student	12 (4.7)	8 (5.3)	Ref	16 (4.8)	4 (5.3)	Ref
	Housewife	196 (76.3)	110 (73.3)	0.59 (0.20–1.73)	248 (74.9)	58 (76.3)	0.62 (0.16–2.35)
	Government employee	33 (12.8)	18 (12)	0.51 (0.14–1.82)	42 (12.7)	9 (11.8)	0.34 (0.07–1.62)
	Private employee	12 (4.7)	11 (7.3)	1.10 (0.28–4.31)	20 (6)	3 (3.9)	0.26 (0.04–1.66)
	None of the above	4 (1.6)	3 (2)	1.22 (0.18–8.3)	5 (1.5)	2 (2.6)	0.98 (0.11–8.48)
Gestational age (weeks)	First trimester	22 (8.6)	5 (3.3)	Ref	22 (6.6)	5 (6.6)	Ref
	Second trimester	59 (23)	32 (21.3)	2.25 (0.73–6.98)	79 (23.9)	12 (15.8)	0.64 (0.18–2.24)
	Third trimester	176 (68.5)	113 (75.3)	3.22 (1.10–9.41)	230 (69.5)	59 (77.6)	1.24 (0.41–3.79)
Gravidity	One	95 (37)	37 (24.7)	Ref	109 (32.9)	23 (30.3)	Ref
	Two	40 (15.6)	27 (18)	1.88 (0.86–4.10)	56 (16.9)	11 (14.5)	1.24 (0.47–3.44)
	More than two	122 (47.5)	86 (57.3)	1.75 (0.71–4.31)	166 (50.2)	42 (55.3)	1.91 (0.64–5.71)
Parity	None	87 (33.9)	38 (25.3)	Ref	101 (30.5)	24 (31.6)	Ref
	Two	69 (26.8)	38 (25.3)	0.94 (0.45–1.97)	89 (26.9)	18 (23.7)	0.63 (0.25–1.59)
	More than two	101 (39.3)	74 (.3)	1.18 (0.45–3.08)	141 (42.6)	34 (44.7)	0.54 (0.16–1.75)
Husband’s occupation	Government employee	173 (67.3)	102 (68)	Ref	225 (68)	50 (65.8)	Ref
	Private employee	47 (18.3)	26 (17.3)	0.86 (0.47–1.56)	57 (17.2)	16 (21.1)	1.82 (0.87–3.79)
	Self-employee	37 (14.4)	22 (14.7)	1.39 (0.68–2.86)	49 (14.8)	10 (13.2)	1.97 (0.79–4.88)
Husband’s educational level	Illiterate	6 (2.3)	0	NAE	6 (1.8)	0	NAE
	Primary school	5 (1.9)	2 (1.3)	NAE	5 (1.5)	2 (2.6)	NAE
	Intermediate school	15 (5.8)	7 (4.7)	NAE	16 (4.8)	6 (7.9)	NAE
	High school	82 (31.9)	60 (40)	NAE	125 (37.8)	17 (22.4)	NAE
	University/College	128 (49.8)	71 (47.3)	NAE	158 (47.7)	41 (53.9)	NAE
	Postgraduate studies	21 (8.2	10 (6.7)	NAE	21 (6.3)	10 (13.2)	NAE
Husband’s income	Low income	24 (9.3)	10 (6.7)	Ref	29 (8.8)	5 (6.6)	Ref
	Lower middle income	210 (81.7)	131 (87.3)	0.90 (0.34–2.36)	278 (84)	63 (82.9)	1.02 (0.27–3.77)
	Upper middle income	23 (8.9)	9 (6)	0.46 (0.12–1.66)	24 (7.3)	8 (10.5)	1.51 (0.31–7.43)

Numbers were presented as frequency (%); NAE: not able to estimate.

## Discussion

The present study examined the pattern of using prescription and nonprescription medications as well as herbal products and supplements during pregnancy among women in the Qassim region in Saudi Arabia. The consumption of prescription medications in the short-term and the usage of nonprescription medications during pregnancy were found in almost 40% of the participants. In addition, the usage of prescription medications for chronic conditions and herbal medications during pregnancy were reported in almost 20% of the participants. In addition, almost all participants were found to be using pregnancy-related medications during their pregnancy. Further analysis revealed that women in their second and third trimesters were more likely to use prescription medications in the short term.

Further, we found that several classes of prescription medications were consumed in the short term during pregnancy; antibiotics were the most frequently utilized medications, followed by analgesics, and antacids. Studies conducted in Switzerland (Ger et al., 2021) and the Netherlands (De Waard et al., 2019) had similar findings. Moreover, the current study found that 4 out of 10 pregnant women used laxatives, compared to 8.7 out of 10 pregnant women in a study conducted in the Netherlands (de Waard et al., 2019). The variation could be attributed to a possibly lower prevalence of constipation within the current research sample. In contrast, the proportion of pregnant women using prescription medicines for chronic illnesses, such as hormone replacement therapy for hypothyroidism, antihypertensive medication for those with hypertension, and antidiabetic medication for those with diabetes were comparable to the proportions observed in research conducted in Norway (Engeland et al., 2018).

According to the findings of the current study, folic acid/iron supplements, and calcium supplements are the most frequently used pregnancy-related medications. These findings are in accordance with a study conducted in Northwest China (Liu et al., 2019), which reported that a higher percentage (>50%) of participants used folic acid and calcium during pregnancy while only 5.4% reported using iron. This similarity may stem from the higher prevalence of higher education among these samples; this emphasizes the participants’ understanding of the need for supplement consumption during pregnancy. Further, the findings of the current study revealed that analgesics were the most frequently utilized nonprescription medications, which is also what was found in another study conducted in Riyadh, Saudi Arabia (Alyami et al., 2023). Most of the participants complained of headaches and fever, leading to the frequent utilization of analgesics. Conversely, in the current study cough/cold medications and antiemetics were much lower than that reported in the Riyadh study (Alyami et al., 2023). This variation could be due to the lower proportion of pregnant women with nausea, cough, or colds in the current research sample. In addition, this study found that a few pregnant women utilized herbal medications—such as those containing ginger, anise, and cumin—to treat certain problems. The findings of this study were comparable to studies conducted in Bangladesh and the United States (Ahmed et al., 2018; Illamol et al., 2020). During the interviews, participants stated that they utilized herbal medication throughout their pregnancy because they believed they were safer than mainstream medicines and are more easily accessible.

The present study also indicated that pregnant women in their second and third trimesters were more likely to use prescription medications in the short term, with antibiotics being the most prescribed medications (62%). This result is in accordance with a multinational study conducted across Europe (Western, Northern, and Eastern), North and South America, and Australia which found that 68% of 9,459 patients used at least one short-term medication during their pregnancy (Lupattelli et al., 2014). This result differs from research conducted in the Netherlands (de Waard et al., 2019), where antibiotics use was noticed in almost 35% of the population. This might be due to restricted antibiotic prescribing in the Netherlands compared to Saudi Arabia. In contrast, pregnant women with more than two previous births were less likely to use prescription medication in the short term during their pregnancy. This result was different from that of a multinational study in Europe, which revealed that women who had previously given birth were more likely to use medication in the short term during their current pregnancy (Lupattelli et al., 2014).

This study has several limitations that should be considered. First, its cross-sectional design captures medication use at a single point in time, which is subject to recall bias and does not allow for monitoring changes in medication patterns throughout the course of a pregnancy. Second, the use of a convenience sample from a single hospital in Buraydah limits the generalizability of the findings to other regions in Saudi Arabia or different healthcare settings, as such samples are vulnerable to various forms of bias (selection, volunteering, and Contextual Bias). Third, the data was collected via self-report questionnaires, which may be influenced by social desirability bias and could not be validated against official medical or pharmacy records. Finally, while the study identified classes of medications, it did not analyze specific drug names, dosages, or the precise timing of use, which are critical for a detailed risk assessment. Additionally, the study did not capture the source of the medication recommendation (e.g., physician, pharmacist, or self-medication), which limits our ability to assess the specific influence of professional pharmacy advice versus lay recommendations.

Despite these limitations, this study’s findings have important implications for clinical practice and public health. First, the safety implications of the observed medication patterns are significant. The high rate of antibiotic use (62.1% of short-term prescriptions) raises concerns regarding antimicrobial resistance (AMR), which may alter the maternal and neonatal microbiome, potentially impacting long-term infant immunity (González-Pérez et al., 2016). Furthermore, the widespread use of OTC analgesics (50% of OTC users) warrants specific caution regarding teratogenicity and fetal safety. While acetaminophen is generally considered safe, the inadvertent use of nonsteroidal anti-inflammatory drugs (NSAIDs) during the third trimester is associated with risks, such as premature closure of the fetal ductus arteriosus and oligohydramnios (Koren et al., 2006). Finally, the use of herbal products containing anise (27.6%) and cumin (18.4%) presents a safety paradox. While users perceive these as safe and natural, robust clinical data establishing their safety profiles and teratogenic risks in medicinal dosages are largely absent. This uncertainty necessitates caution and highlights the urgent need for evidence-based education regarding the unsupervised use of “natural” products (Bernstein et al., 2020).

Consequently, strict adherence to global safety guidelines is necessary to mitigate these risks. Our findings revealed that antibiotics were the most frequently used prescription medication for short-term conditions, primarily for UTIs. This aligns with ACOG guidelines, which emphasize that while antibiotic stewardship is crucial, untreated bacteriuria in pregnancy carries significant risks, such as pyelonephritis and preterm birth, thereby necessitating appropriate treatment (ACOG, 2023). However, the widespread use of antibiotics also emphasizes the need for enhanced pharmacovigilance to monitor prescribing patterns and enforce the Saudi Ministry of Health’s National Action Plan on Antimicrobial Resistance, which is in line with WHO recommendations for rational drug use during pregnancy (Zanichelli et al., 2023; Sheerah et al., 2025). With regard to herbal remedies, our study found that ginger was most commonly (44.7%) used primarily for nausea. This practice is supported by ACOG Practice Bulletin No. 189 on nausea and vomiting in pregnancy, which considers ginger a reasonable nonpharmacological option for mild symptoms (ACOG Practice Bulletin No. 189, 2018). Conversely, the near-universal use of folic acid (98.3%) and iron (92.0%) in our cohort reflects strong adherence to Saudi Ministry of Health antenatal protocols and WHO antenatal care guidelines (Tunçalp et al., 2017; Alsolami et al., 2024).

From a pharmacovigilance perspective, the concurrent use of prescription drugs, OTC medications (reported by 36.9% of participants), and herbal products highlights a complex safety profile that requires active management. Therefore, healthcare providers, particularly clinical pharmacists, must act as the primary checkpoint for medication safety. Implementing routine medication reconciliation processes during antenatal visits is essential to identify potential drug–herb interactions and ensure that all therapeutic interventions—whether prescribed or self-administered—adhere to the safety standards outlined by WHO and ACOG (Cheema et al., 2018). To operationalize this, we propose implementing active pharmacist-led verification, such as “brown bag” reviews, to comprehensively assess all patient-consumed products (Dunston et al., 2025). Furthermore, targeted prenatal education modules are needed to address specific knowledge gaps regarding herbal safety and trimester-specific NSAID avoidance.

Future research should aim to overcome the limitations of this study. Prospective, multicenter cohort studies across different regions of Saudi Arabia are needed to provide a more dynamic and representative picture of medication use. Such studies should validate self-reported data against medical and prescription records to ensure accuracy. Furthermore, qualitative research could provide valuable insights into the beliefs and attitudes that drive medication choices among pregnant women, particularly regarding herbal remedies. Finally, linking medication exposure data to maternal and neonatal health outcomes would be invaluable for establishing the safety profiles of the most commonly used medications in this population.

## Conclusion

This study’s findings highlight that a considerable number of pregnant women use a variety of medications during their pregnancy. The medications that were most frequently used for acute/short-term illnesses were antibiotics, while the ones for chronic/long-term conditions were those used for hormonal replacement therapy. During pregnancy, common OTC medications, and herbal remedies, such as analgesics and antacids, were frequently utilized. Additionally, pregnant women were found to commonly take additional supplements such as folic acid as well as various vitamins and minerals. The common reasons for using medications during pregnancy were UTI, hypothyroidism, headaches, and nausea.

## Data Availability

The raw data supporting the conclusions of this article will be made available by the authors, without undue reservation.
