# Supplementary material for: Patterns of prescription, over-the-counter, and herbal medication use among pregnant women in Buraydah, Saudi Arabia: a cross-sectional study
Source: Front Pharmacol. 2026 Jan 2;16:1732619. doi: 10.3389/fphar.2025.1732619 (PMC12808483; doi:10.3389/fphar.2025.1732619)
Supplement: Supplementary file 1 [file DataSheet1.pdf]

Appendix-A: questionnaire

**Demographic characteristics**	
**1. Age in years**<br>☐ < 25 years old<br>☐ 25-29 years old<br>☐ 30-34 years old<br>☐ ≥ 35 years old	**2. Place of residence**<br>☐ Urban<br>☐ Rual
**3. Educational level**<br>☐ Illiterate<br>☐ Primary school<br>☐ Intermediate school<br>☐ High school<br>☐ University/College<br>☐ Postgraduate studies	**4. Occupation**<br>☐ Student<br>☐ Housewife<br>☐ Government employee<br>☐ Private employee<br>☐ None of the above
**5. Gestational age (weeks)**<br>☐ First trimester<br>☐ Second trimester<br>☐ Third trimester	**6. Gravidity (no. of previous pregnancies)**<br>☐ One<br>☐ Two<br>☐ More than two
**7. Parity (no. of previous live births)**<br>☐ None<br>☐ Two<br>☐ More than two	**8. Husband's educational level**<br>☐ Illiterate<br>☐ Primary school<br>☐ Intermediate school<br>☐ High school<br>☐ University/College<br>☐ Postgraduate studies
**9. Husband's occupation**<br>☐ Government employee<br>☐ Private employee<br>☐ Self-employee	**10. Husband's income**<br>☐ Low income<br>☐ Lower middle income<br>☐ Upper middle income

Drug use characteristics

1. **Did you use any prescription medication for short-term?** (i.e., Nausea, vomiting, UTI, inflammation, heartburn, headache, constipation, pain, etc.)
   - ☐ Yes
   - ☐ No

If yes, please complete the following questions:

- What is the name of the medicine? ....
- What is the reason for use (indication):……………………………………..
- What is the period of use (pregnancy weeks):…………………………………

2. **Did you use any prescription medication for chronic conditions?** (i.e., Hypothyroidism, asthma, allergy, hypertension, diabetes, cardiac disease, cancer, depression, osteoporosis, etc.)
    - ☐ Yes
    - ☐ No

If yes, please complete the following questions:

- What is the name of the medicine? ....
- What is the reason for use (indication):………………………………………..
- What is the period of use (pregnancy weeks):…………………………………

3. **Did you use any pregnancy-related drugs?** (i.e., Vitamins, Folic acid, etc.)
    - ☐ Yes
    - ☐ No

If yes, please complete the following questions:

- What is the name of the medicine? ....
- What is the reason for use (indication):………………………………………..
- What is the period of use (pregnancy weeks):…………………………………

4. **Did you use any nonprescription medications during pregnancy?** (i.e., Nausea, vomiting, UTI, inflammation, heartburn, headache, constipation, pain, etc.)
    - ☐ Yes
    - ☐ No

If yes, please complete the following questions:

- What is the name of the medicine? ....
- What is the reason for use (indication):………………………………………..
- What is the period of use (pregnancy weeks):…………………………………

5. **Did you use any herbal medicine during pregnancy?**
    - ☐ Yes
    - ☐ No

If yes, please complete the following questions:

- What is the name of the medicine? ....
- What is the reason for use (indication):………………………………………..
- What is the period of use (pregnancy weeks):…………………………………
